# A Rare Case of Aggressive Peripheral T-cell Lymphoma–Not Otherwise Specified

**DOI:** 10.7759/cureus.24857

**Published:** 2022-05-09

**Authors:** Ateeb Ur Rahman, Amna Chaudary, Sonia Varandani

**Affiliations:** 1 Internal Medicine, WellSpan York Hospital, York, USA; 2 Internal Medicine, Drexel University College of Medicine, Philadelphia, USA

**Keywords:** prognosis of peripheral t cell lymphoma, hematological malignancy, non-hodgkin's lymphoma, not otherwise specified, peripheral t cell lymphoma

## Abstract

Peripheral T-cell lymphomas are an offshoot of non-Hodgkin’s lymphomas and usually carry a poor prognosis. Their clinical manifestations could be very variable and can mimic an infectious or autoimmune etiology.

Here, we present a case of a 58-year-old healthy female who presented with fever, cough, and shortness of breath for several days. Imaging studies including chest x-ray and CT scans were suggestive of pleural effusions, mediastinal/retroperitoneal lymphadenopathy, and splenomegaly. She was initially managed for severe sepsis in the setting of possible community-acquired pneumonia. Later her course of hospitalization was complicated by respiratory failure and needing mechanical ventilation and then extracorporeal membrane oxygenation (ECMO). Multiple biopsies were performed including bone marrow and lymph nodes which were suggestive of peripheral T-cell lymphoma - not otherwise specified. Due to the severity of her illness, palliative discussions were made and the family opted for comfort care.

## Introduction

Peripheral T-cell lymphoma (PTCL) accounts for 6%-10% of all non-Hodgkin lymphomas in the world, which makes it exceedingly rare [1]. Among the different types of T cell lymphomas, Peripheral T-cell lymphoma-not otherwise specified (PTCL-NOS) accounts for almost 25% of cases worldwide [2]. Due to their insidious onset, aggressive nature, and limited treatment options, PTCLs, in general, have poor outcomes [2]. PTCL indicates a lymphoma of mature T lymphocytes. Mature T lymphocytes are often referred to as “post-thymic” or “peripheral” [3]. While PTCL is often classified as leukemic/disseminated, nodal, extra-nodal, or cutaneous based on the predominant features, the heterogeneous presentation can sometimes result in the classification of not otherwise specified (NOS) [3]. This is an unusual case of a patient found to have this rare hematological malignancy.

## Case presentation

**Figure 1:**
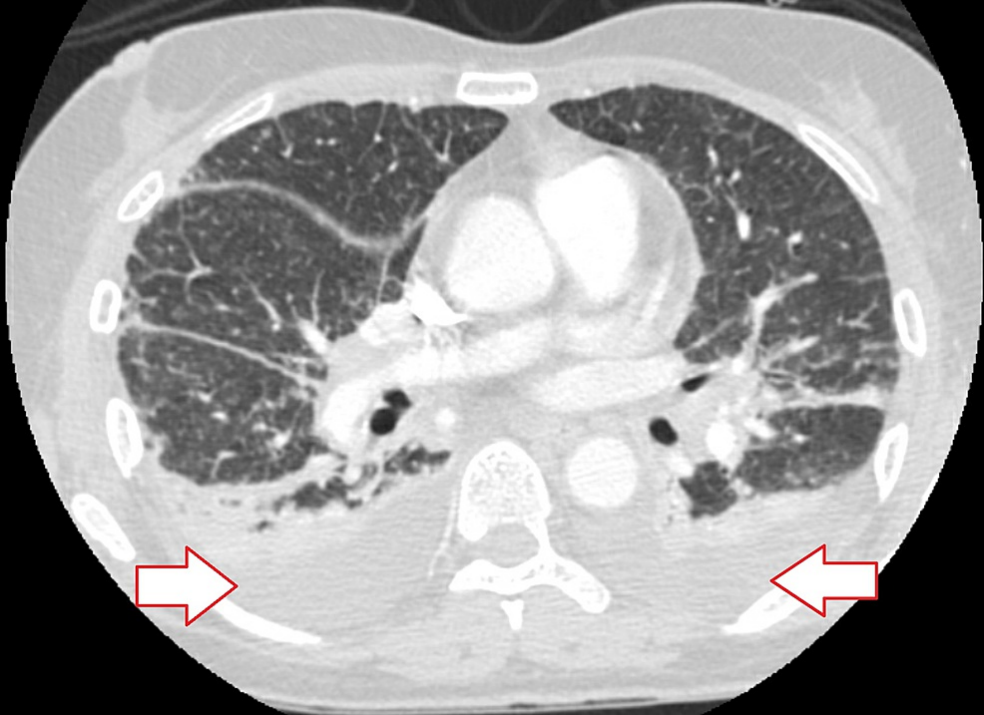
Arrows point to bilateral pleural effusions

The patient initially presented to the hospital with complaints of fever, worsening dyspnea, and cough for several days. Chest x-ray and CT chest (Figure 1) initially showed signs suggestive of bilateral pneumonia and parapneumonic pleural effusions.

**Figure 2:**
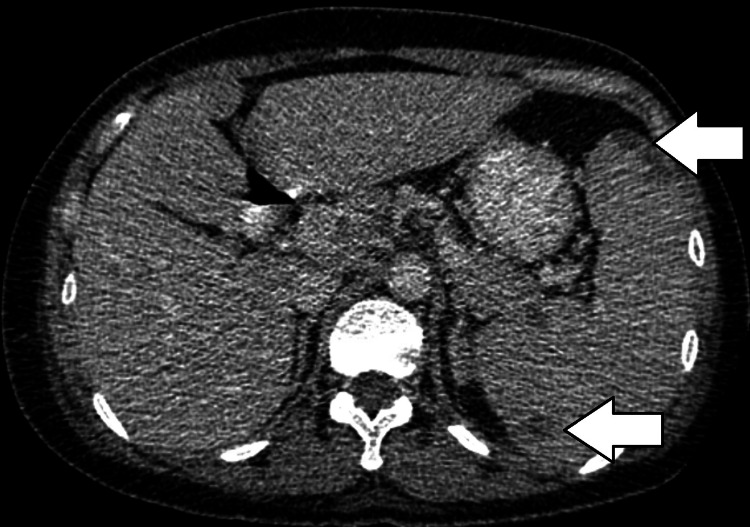
Arrows point to multiple hypodense lesions in the spleen

**Figure 3:**
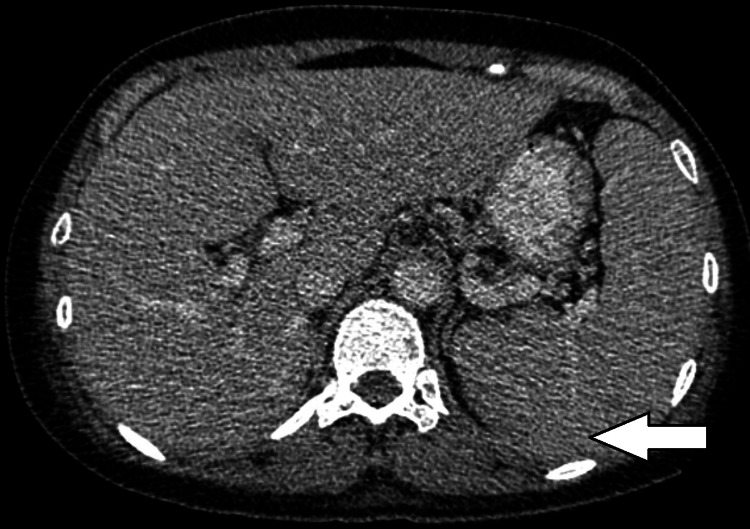
Arrow points to enlarged spleen

Initial treatment was started with antibiotics for community-acquired pneumonia and an extensive workup was performed to rule out other etiologies of her symptoms. The hospital stay was prolonged with multiple fevers with a T max of 106.6 Fahrenheit, worsening oxygenation, and persistently elevated inflammatory markers despite multiple negative COVID-19 tests, blood, and pleural fluid cultures. She completed several courses of antibiotics without any significant improvement of symptoms. The patient eventually started to develop profound anemia and thrombocytopenia. Due to worsening oxygenation and hemodynamic instability, the patient was intubated and transferred to the ICU. She eventually developed multiorgan failure required maximum oxygen support and was placed on extracorporeal membrane oxygenation (ECMO). Significant imaging studies during hospitalization showed mediastinal and hilar adenopathy, retroperitoneal lymphadenopathy, multiple hypodensities (Figure 2), and splenomegaly (Figure 3) without the ability to exclude lymphoma.

**Figure 4:**
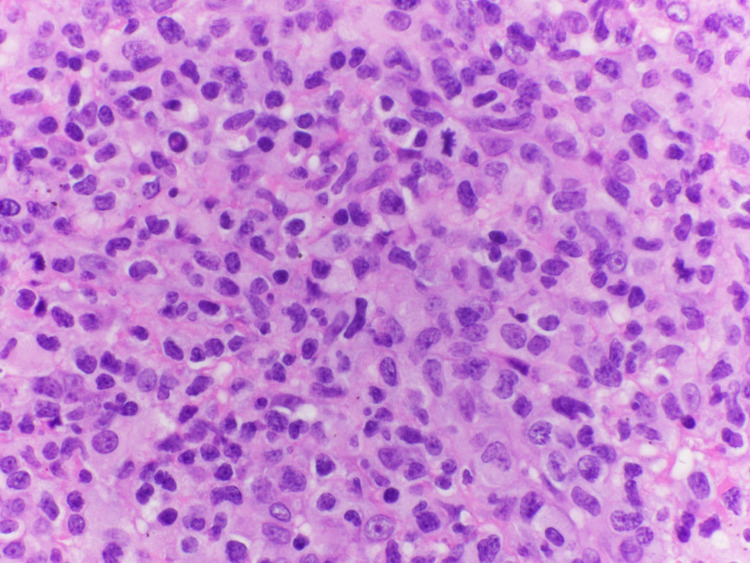
Large tumor cells with nuclear atypia and prominent nucleoli

**Figure 5:**
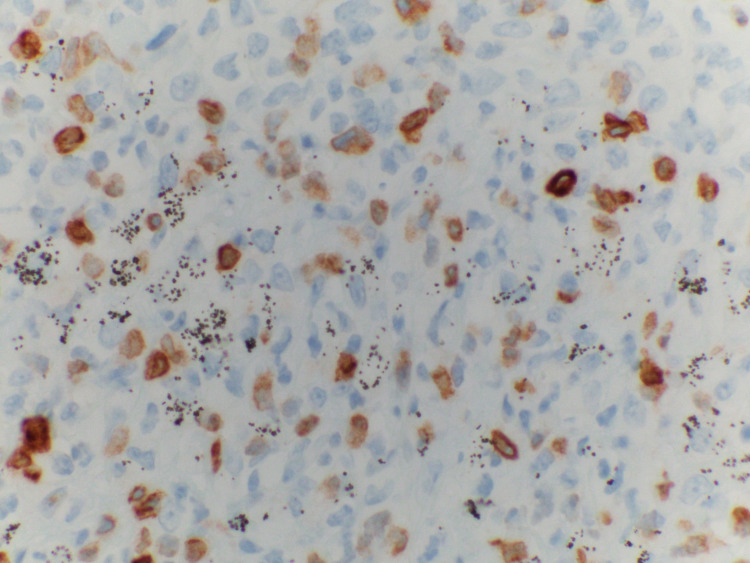
Partial CD-30 expression on Immunohistochemistry CD: Cluster of Differentiation

The patient had multiple peripheral blood smears which were nonconclusive early on. A bone marrow biopsy and a bronchoscopy were performed afterward and those were non-diagnostic too. Due to the patient's worsening clinical condition, it was decided to pursue a VATS (video-assisted thoracoscopic surgery) with lymph node dissection and a repeat bone marrow biopsy. Finally, the diagnosis was made with a combination of a biopsy of lymph nodes, pleural fluid analysis, and a repeat bone marrow biopsy. The analysis included histopathological findings (Figure 4) in combination with flow cytometry and Immunohistochemistry (Figure 5), which was suggestive of PTCL-NOS.

The patient was quickly started on Gemcitabine and Oxaliplatin, but five days after chemotherapy the patient continued to decline and was found to have metastatic lesions in the brain. After a family discussion, the patient was transitioned to comfort care and died soon after the withdrawal of care.

## Discussion

T-cell lymphomas are aggressive cancers and ones that can have an insidious presentation and are generally associated with poor clinical outcomes. PTCL-NOS is the most common type of PTCLs. The incidence ratio in males to females is 2:1, and the median age of presentation is 62 years [3]. PTCLs are more prevalent in Asia, due to the geographic variability of different viral exposures such as the Ebstein-Barr virus (EBV) or human T-lymphotropic virus 1 (HTLV-1) [3,4]. Albeit, these aggressive lymphomas are also found in the western nations and account for almost 5%-10% of all non-Hodgkin lymphomas [3]. PTCL-NOS is usually found in the lymph node but can have extranodal manifestations as well in the gastrointestinal tract or skin [4].

Five-year overall survival for PTCL-NOS is 20%-32% generally because their indolent forms are far and few and usually aggressive in nature [2,4]. Even when compared with aggressive B-cell lymphomas, PTCLs have bad outcomes [2]. The prognosis of PTCL-NOS depends on multiple factors but mainly, on molecular profiling and cytogenetics [2]. Current molecular advancements have increased the potential to identify prognostic biomarkers for risk-stratification of patients, which can help guide treatment [1,3]. It is considered to have an adverse prognostic impact if PTCL-NOS has a high expression of GATA3, or TBX21 [4]. The current prognostic index for PTCL-NOS includes age, lactate dehydrogenase (LDH) levels, and extent of bone marrow involvement [5,6]. It has been reported that the neutrophil to lymphocyte ratio can be used as a prognostic indicator in PTCL-NOS [6].

Treatment options that are currently available include chemotherapy, immunomodulators, histone deacetylase (HDAC) inhibitors, and signal blockers but with very limited efficacy [2,4]. Given the rare nature of PTCL-NOS, many management options have been derived from treatments for different neoplasms, such as non-Hodgkin’s B-cell neoplasms. Developing treatment options that target PTCL-NOS specifically is key to improving the prognosis[7]. Feldman et al reported SYK overexpression in a subset of PTCL-NOS patients in one study along with a detectable interleukin-2 inducible T-cell kinase-spleen tyrosine kinase (ILK-SYK) fusion gene translocation t(5;9). This indicates a potential therapeutic approach with SYK inhibitors [5]. The role of brentuximab vedotin (a potent CD-30 inhibitor) is being studied for PTCL-NOS either alone or with chemo or stem cell transplant [8].

## Conclusions

This case highlights the importance of timely diagnosis in such aggressive lymphomas. The patient had multiple negative peripheral smears, a bone marrow biopsy, and an inconclusive bronchoscopy. Without the appropriate diagnosis, it is extremely difficult to analyze the effect of PTCL-NOS. Therefore, in case of high clinical suspicion, it is important that the utilization of a repeat bone marrow biopsy or lymph node sampling should be done. Despite the advent of novel treatment options for PTCL-NOS, progression-free survival has still not improved significantly. With the advancement in science and a better understanding of T-cell lymphomas with molecular and genomic stratifications, we anticipate more timely and effective management of PTCL-NOS is on the horizon.
